# Skin Inflammation—A Cornerstone in Dermatological Conditions

**DOI:** 10.3390/jpm12091370

**Published:** 2022-08-25

**Authors:** Mircea Tampa, Monica Neagu, Constantin Caruntu, Carolina Constantin, Simona Roxana Georgescu

**Affiliations:** 1 Department of Dermatology, “Carol Davila” University of Medicine and Pharmacy, 020021 Bucharest, Romania; 2 Dermatology Department, “Victor Babes” Hospital of Infectious Diseases, 030303 Bucharest, Romania; 3 “Victor Babes” National Institute of Pathology, 050096 Bucharest, Romania; 4 Colentina University Hospital, 020125 Bucharest, Romania; 5 Department of Physiology, “Carol Davila” University of Medicine and Pharmacy, 050474 Bucharest, Romania

The skin provides more than a simple mechanical barrier against external aggressors; it is an array of effector cells and molecules that constitute the skin’s immune system, as defined by Bos and Kapsenberg in 1986 [1]. There are many types of cells that contribute to the immune function of the skin. It is believed that there are 20 billion lymphocytes in the layers of the skin, which probably explains the crucial role of these cells in various pathologies. In the epidermis, the dominant immune cell populations are represented by Langerhans cells and CD8+ tissue-resident memory T cells, and in the dermis, there are dendritic cells, macrophages, innate lymphoid cells, natural killer cells, and CD8+ tissue-resident memory T cells. Memory T cells seem to play an important role in the pathogenesis of inflammatory skin diseases (ISDs) [2]. It should be considered that most ISDs are defined as T-cell-mediated diseases. In the last decade, researchers have focused on the study of Th17 cells in the skin. Th17 cells are involved in the defense against extracellular pathogens and in autoimmune processes. Psoriasis is arguably the best-characterized skin condition related to this cell subset [3]. Important progress has been made in understanding the functions of the numerous cells found in the skin and the communication pathways between them. However, the triggering factors that disrupt the local immune homeostasis and generate the cascade of inflammation in many ISDs remain unclear. Additional studies are needed to improve our understanding of how the balance between proinflammatory and anti-inflammatory systems is altered.

Since ISDs represent a heterogeneous group of diseases, they are most often analyzed as separate entities. ISDs are characterized by an abnormal response of immune cells to various endogenous or exogenous stimuli, with the initiation and perpetuation of an inflammatory process that, in most cases, becomes chronic. ISDs are complex conditions that involve the interplay of numerous factors such as genetic, infectious, immune, lifestyle, and environmental factors, leading to different clinical manifestations that most frequently include erythema, scales, or itching. The pathogenic mechanisms of ISDs still represent a challenge in modern medicine despite the remarkable progress achieved in recent years. Chronic ISDs are often associated with physical and mental distress; therefore, they have important impacts on patients’ quality of life. In recent decades, extensive research has focused on unraveling the mechanisms underlying these conditions. Six immune-response patterns of the skin were recently discussed (lichenoid, eczematous, bullous, psoriatic, fibrogenic, and granulomatous patterns) [4]. Atopic dermatitis and psoriasis represent some of the most-studied chronic ISDs. A recent study focusing on the two conditions highlights the role of the epithelial immune microenvironment, including the immune and non-immune cells from the epidermis and superficial dermis, along with the skin microbiota [5]. An increasing number of studies are exploring the skin microbiota, taking into account the recent remarkable achievements in the field of bacterial identification techniques. Knowing the characteristics of the skin microbiome and its interactions with the innate and adaptive immune systems can lead to a better understanding of ISDs and to novel therapeutic approaches. Although numerous studies have highlighted changes in the microbial flora in patients with various dermatoses, it is not clear if these alterations play a role in the pathogenesis or are the result of the inflammatory process. This topic remains open for new research and the clarification of these unknown variables [6].

In the last decade, the study of oxidative stress has become a hot topic with regard to numerous conditions such as cardiovascular, pulmonary, neurodegenerative, and infectious diseases, as well as skin diseases. Analyzing the effects of alterations in the balance between oxidants and antioxidants in the skin, with important impacts on signaling pathways and cell proliferation, differentiation, and apoptosis, could open new avenues in the methods of diagnosis and therapy for ISDs. A growing body of evidence indicates the involvement of oxidative stress in chronic ISDs such as psoriasis, atopic dermatitis, vitiligo, and alopecia areata. The dilemma is whether oxidative stress is the initiator of chronic inflammatory diseases or is the result of the interaction between inflammatory cells and the proinflammatory cytokines. In the skin, oxidative stress initiates an inflammatory process that can become chronic through the accumulation and persistence of reactive oxygen species, leading to clinical signs such as erythema, edema, and pain.

Although there is no clear evidence, autoimmunity is a component in the pathogenesis of many ISDs that requires further exploration. In addition to diseases in which the autoimmune process is relatively well established, such as vitiligo, pemphigus or bullous pemphigoid, recent research indicates a role for autoimmunity in diseases such as psoriasis or atopic dermatitis. Alteration of the skin barrier seems to be closely related to autoimmune events. Thus, changes in the cell components can represent a triggering factor for pathogenic adaptive autoimmune reactions. Several factors can lead to cell damage including UV radiation, trauma, and infections. Necrotic cells represent an important source of antigens that reach the extracellular space and can elicit an immune response. This mechanism has been described, in particular, in autoimmune blistering diseases of the skin [7].

Chronic inflammation is related to regulatory T cells, Th2 cells, and mediators of inflammation such as transforming growth factor beta (TGF)-β, interleukin (IL)-4, IL-6, IL-10, and IL-13. Molecules such as TGF-β, IL-6, and IL-10 act as protumorigenic mediators inducing tumor onset and progression. Therefore, a chronic inflammatory process creates an environment favorable for carcinogenesis [8]. The pathogenesis of the most common skin cancers, basal cell and squamous cell carcinomas, is closely related to inflammation. UV radiation, one of the main risk factors for the development of skin cancers, induces DNA damage and generates oxidative stress, two events that trigger inflammatory processes. Chronic inflammation and immune responses are key elements that define the tumor microenvironment, which encompasses a myriad of inflammatory and immune cells, tumor cells, and signaling molecules that form an immunoregulatory network able to induce an immunosuppressive state favoring tumor progression, invasion, and metastasis [9].

The diagnosis of ISDs is generally based on the patient’s medical history and clinical examinations, but the gold standard for diagnosis remains the skin biopsy. However, in some cases, the histopathological examination is inconclusive due to the similarity between these diseases. Furthermore, biopsy is not always preferred for various reasons, such as aesthetic concerns or abnormal wound healing. In recent years, more and more studies have evaluated the usefulness of imaging techniques such as reflectance confocal microscopy, optical coherence tomography, and multiphoton microscopy in the diagnosis of ISDs. They are non-invasive and rapid techniques aimed at improving the diagnosis of ISDs. However, an analysis of existing studies shows a lack of data on their use in current practice; further research is required to establish the correct parameters for the diagnosis, staging, and assessment of the response to therapy of ISDs [10].

The medical literature is abounding with studies that focus on the assessment of circulating biomarkers in the blood of patients with skin diseases. Directing attention to the study of reliable biomarkers could lead to the identification of better diagnostic tools, and the standardization of laboratory methods could improve the management of patients with skin diseases. As aforementioned, ISDs are associated with alterations in the molecular components of the skin. Thus, the analysis of skin chemistry (lipids, proteins, nucleic acids, and molecules involved in inflammation) could also represent an important source of biomarkers for the diagnosis of these conditions and the evaluation of the effectiveness of various therapies, but the research in this field has only just begun [11].

Corticosteroids have been the basic therapy for inflammatory diseases for years. However, ISDs represent a heterogeneous group of diseases, and patients differ according to their genetic backgrounds. Therefore, better and more targeted treatments are needed. Understanding the mechanisms underlying the pathogenesis of chronic ISDs has led to the development of biological therapies that have dramatically changed the management of conditions such as psoriasis and atopic dermatitis, considerably improving patients’ quality of life. Biological therapies inhibit the inflammatory response within the skin by hindering the activation of signaling pathways and the release of proinflammatory cytokines. Biologics targeting TNF-α, IL-12/23, IL-17, and IL-23 have been commercialized for the management of psoriasis, and many other molecules are being evaluated in various clinical trials. For atopic dermatitis, a monoclonal antibody that blocks IL-4R, dupilumab; a phosphodiesterase (PDE4) inhibitor, crisaborole; and newly approved Janus kinase inhibitors have changed the approach to patients with severe forms of the disease [12]. Recent research has focused on Janus kinase inhibitors, a class of drugs that also show immense promise in the treatment of diseases other than atopic dermatitis such as alopecia areata and vitiligo.

Although the five cardinal signs of inflammation (calor, dolor, rubor, tumor and functio laesa) have been described since ancient times, there are still many questions surrounding the inflammatory process. The triggering factors, the signaling pathways, and the roles of the molecules and cells involved remain to be fully elucidated, even today. ISDs represent a puzzle with many missing pieces, but we know that inflammation remains the cornerstone of their pathogenesis; starting from here, we should explore further to solve the puzzle. Owing to the importance of the ISDs, our Special Issue will cover all aspects of these complex skin pathologies.
